# Perfusion-weighted software written in Python for DSC-MRI analysis

**DOI:** 10.3389/fninf.2023.1202156

**Published:** 2023-08-01

**Authors:** Sabela Fernández-Rodicio, Gonzalo Ferro-Costas, Ana Sampedro-Viana, Marcos Bazarra-Barreiros, Alba Ferreirós, Esteban López-Arias, María Pérez-Mato, Alberto Ouro, José M. Pumar, Antonio J. Mosqueira, María Luz Alonso-Alonso, José Castillo, Pablo Hervella, Ramón Iglesias-Rey

**Affiliations:** ^1^Neuroimaging and Biotechnology Laboratory (NOBEL), Clinical Neurosciences Research Laboratory (LINC), Health Research Institute of Santiago de Compostela (IDIS), Santiago de Compostela, Spain; ^2^Centro de Supercomputación de Galicia (CESGA), Santiago de Compostela, Spain; ^3^Nasasbiotech S. L., A Coruña, Spain; ^4^Translational Stroke Laboratory (TREAT), Clinical Neurosciences Research Laboratory (LINC), Health Research Institute of Santiago de Compostela (IDIS), Santiago de Compostela, Spain; ^5^Neurological Sciences and Cerebrovascular Research Laboratory, Department of Neurology and Stroke Center, La Paz University Hospital, Neuroscience Area of IdiPAZ Health Research Institute, Universidad Autónoma de Madrid, Madrid, Spain; ^6^NeuroAging Group (NEURAL), Clinical Neurosciences Research Laboratory (LINC), Health Research Institute of Santiago de Compostela (IDIS), Santiago de Compostela, Spain; ^7^Centro de Investigación Biomédica en Red en Enfermedades Neurodegenerativas (CIBERNED), Instituto de Salud Carlos III, Madrid, Spain; ^8^Department of Neuroradiology, Hospital Clínico Universitario, Health Research Institute of Santiago de Compostela (IDIS), Santiago de Compostela, Spain

**Keywords:** DSC-MRI imaging, glioblastoma (GBM), neuroimaging, perfusion analysis, Python, stroke

## Abstract

**Introduction:**

Dynamic susceptibility-weighted contrast-enhanced (DSC) perfusion studies in magnetic resonance imaging (MRI) provide valuable data for studying vascular cerebral pathophysiology in different rodent models of brain diseases (stroke, tumor grading, and neurodegenerative models). The extraction of these hemodynamic parameters via DSC-MRI is based on tracer kinetic modeling, which can be solved using deconvolution-based methods, among others. Most of the post-processing software used in preclinical studies is home-built and custom-designed. Its use being, in most cases, limited to the institution responsible for the development. In this study, we designed a tool that performs the hemodynamic quantification process quickly and in a reliable way for research purposes.

**Methods:**

The DSC-MRI quantification tool, developed as a Python project, performs the basic mathematical steps to generate the parametric maps: cerebral blood flow (CBF), cerebral blood volume (CBV), mean transit time (MTT), signal recovery (SR), and percentage signal recovery (PSR). For the validation process, a data set composed of MRI rat brain scans was evaluated: i) healthy animals, ii) temporal blood–brain barrier (BBB) dysfunction, iii) cerebral chronic hypoperfusion (CCH), iv) ischemic stroke, and v) glioblastoma multiforme (GBM) models. The resulting perfusion parameters were then compared with data retrieved from the literature.

**Results:**

A total of 30 animals were evaluated with our DSC-MRI quantification tool. In all the models, the hemodynamic parameters reported from the literature are reproduced and they are in the same range as our results. The Bland–Altman plot used to describe the agreement between our perfusion quantitative analyses and literature data regarding healthy rats, stroke, and GBM models, determined that the agreement for CBV and MTT is higher than for CBF.

**Conclusion:**

An open-source, Python-based DSC post-processing software package that performs key quantitative perfusion parameters has been developed. Regarding the different animal models used, the results obtained are consistent and in good agreement with the physiological patterns and values reported in the literature. Our development has been built in a modular framework to allow code customization or the addition of alternative algorithms not yet implemented.

## 1. Introduction

Dynamic susceptibility-weighted contrast-enhanced (DSC) perfusion studies in magnetic resonance imaging (MRI) provide valuable data for studying vascular cerebral pathophysiology. It is possible to acquire functional information about perfusion-related parameters such as cerebral blood flow (CBF), mean transit time (MTT), and cerebral blood volume (CBV) (Keston et al., 2003; Østergaard, 2004). The accurate quantification of these parameters has several clinical applications, including the identification and evaluation of ischemic stroke prior to treatment (Calamante et al., 2002; Callewaert et al., 2021), the description of lesions associated with multiple sclerosis (Haselhorst et al., 2000; D'haeseleer et al., 2015), the diagnosis of tumors (Stadlbauer et al., 2015; Choi et al., 2016), or as trackers of Alzheimer's disease progression (Lacalle-Aurioles et al., 2014; Warpechowski et al., 2023).

This imaging technique is based on monitoring MRI signal strength variations after the injection of a bolus of a paramagnetic contrast agent, such as gadolinium salt-diethylenetriaminepentaacetic acid (Gd DTPA). On T2^*^-weighted images, the circulation of the bolus results in a decrease in signal intensity due to small variations in the local magnetic field, displaying, as a result, the time course of this tracer through the tissue. In clinical practice, there are different available software to process these images, contrary to preclinical studies. Nowadays, rodent models of human brain disorders represent more than 80% of the animals in research. In this sense, *in vivo* perfusion by MRI in rodents is applied to monitor disease progressions, such as stroke, tumor size, neurodegeneration, and evaluation of therapeutic response in longitudinal studies, as well as to develop new animal models of different pathologies (Boisserand et al., 2017; Park et al., 2020; Qi et al., 2021). Therefore, it would be appealing to have a tool capable of performing the whole hemodynamic quantification process in a fast and reliable way for research purposes.

On the technical side, the extraction of these hemodynamic parameters via DSC-MRI is based on tracer kinetic modeling, which can be solved using deconvolution-based methods, among others. Most of the post-processing software used in preclinical studies are home-built and custom-designed, its use being, in most cases, limited to the institution responsible for the development. While there do exist some commercial software tools, they are expensive and specifically designed for clinical use (Gordaliza et al., 2015; López-Larrubia, 2018; Hartmann et al., 2020; Tsai et al., 2021). These tools often only provide relative values (no absolute tissue hemodynamic parameters), do not compute all of the main perfusion parameters (CBF, CBV, and MTT), and overlook direct parameters such as signal recovery (SR) and percentage signal recovery (PSR), which may add valuable diagnostic information without requiring additional measurements (Huhndorf et al., 2016). These restrictions do not allow direct comparison with other software, highlighting the need for an open-source implementation of a DSC-MRI perfusion software application for use in preclinical studies. Furthermore, making it free and open-access could help toward the standardization of DSC-MRI methodology, a pressing issue limiting this method's potential (Boxerman et al., 2020).

In this study, we present the implementation of an open-source DSC quantification tool, named Perfusion-NOBEL, developed as a Python project. Our semi-automatic approach requires no pre-processing aside from the manual delineation of masks, it provides absolute perfusion maps (CBF, CBV, MTT, including SR, and PSR), and it was validated on a large and diverse data set composed of 30 MRI rat brain scans of different models of brain diseases. These data set included i) healthy animals, ii) temporal blood–brain barrier (BBB) dysfunction animals, iii) cerebral chronic hypoperfusion (CCH) model, iv) ischemic stroke model, and v) glioblastoma multiforme (GBM) model. In addition, the resulting hemodynamic parameters were then compared with the literature data. Our tool has been built in a modular framework to allow code customization and the addition of alternative algorithms not yet implemented.

## 2. Materials and methods

### 2.1. Theoretical basis and mathematical description

Quantitative analysis of tissue perfusion and blood volume was performed using established tracer kinetic models, which have been extensively reviewed in the literature (Ostergaard et al., 1996; Wu et al., 2003). In brief, the mathematical approach to this process starts from the linear relation between the T2^*^ relaxation time variation [transverse relaxation rate change ($R_{2}^{*}\left(t\right)$)], signal intensity change, and the concentration of the contrast agent. These relations go as follows:


(1) $$\;\begin{array}{c} S\left(t\right)=S_{0}e^{-TE\cdot R_{2}^{*}(t)}\\ \Delta R_{2}^{*}(t)=k\cdot C_{m}(t) \end{array}\}\;\;\Rightarrow C_{m}\left(t\right)=\;-\frac{k}{TE}\;\ln\left(\frac{S\left(t\right)}{S_{0}}\right)$$

*C*_*m*_(*t*) is the concentration of contrast agent measured in the tissue, TE is the echo time, *S*(*t*) is the signal intensity at a given time, *S*_0_ is the signal intensity before the contrast agent injection, and *k* is a proportionality constant which, to a first approximation, does not depend on the size and geometry of the vessel, or in the density of the capillary bed.

The tissue response to the arrival of the contrast agent is very sensitive to many factors such as the bolus arrival time and the vascular system, causing the delay and dispersion of the bolus injected. In order to obtain an accurate quantification taking into account these effects, an arterial input function (AIF) is used, measuring the concentration of contrast agent arriving at the vessel. The use of arteries as reference vessels is widely documented in the literature, but it is also possible to use veins [venous output function (VOF)]. The AIF is semi-manually obtained from the DSC-MRI images by delineating and masking the desired region.

In terms of system analysis, the measured concentration curve, *C*_*m*_(*t*), the output (response of the tissue to the injection of contrast agent), is related to the ideal concentration curve with no delay or dispersion, *k*(*t*), the input, through the convolution of the former with the AIF, the impulse response function, as shown in the following equation:


(2) $$\begin{array}{l} C_{m}\left(t\right)=k\left(t\right)*C_{AIF}\left(t\right) \end{array}$$

The ^*^ represents the convolution and it can be interpreted as the *C*_*AIF*_(*t*) modifying the shape of the ideal concentration curve *k*(*t*), to give the measured concentration curve *C*_*m*_(*t*). Through the inverse operation, known as deconvolution, it is possible to obtain *k*(*t*), needed for the following analysis, given that both *C*_*m*_(*t*) and *C*_*AIF*_(*t*) are known. This ideal concentration curve, *k*(*t*), it can be also referred as flow-scaled residue function (Fieselmann et al., 2011).

An optional step before the deconvolution process can be taken by fitting the curve *C*_*AIF*_(*t*) to a gamma variate function (Calamante et al., 2000):


(3) $$C_{AIF}\left(t\right)=\{\begin{array}{cc} 0, & t\leq 0\\ C_{0}{\left(t-t_{0}\right)}^{r}\;e^{-\frac{t-t_{0}}{b}},\; & t>0 \end{array}$$

where *t*_0_ is the bolus arrival time (BAT) and determines the arrival of the bolus to any given region. This step helps to reduce noise and avoid other effects such as the recirculation of the contrast agent.

The deconvolution step can be computed using different methods. Truncated single value decomposition (TSVD) and Tikhonov regularization are two of these methods, both being appropriate for the resolution of ill-posed problems like this one (Calamante et al., 2000; Fieselmann et al., 2011). In our program, we have implemented both methods of resolution. Then, the quantitative perfusion parameters, CBF, CBV, and MTT, can be obtained from the concentration curves resulting.

CBF and CBV are computed using deconvolution (Fieselmann et al., 2011):


(4) $$\begin{array}{l} CBF\;\left[\frac{ml}{100g\cdot \min}\right]\\ =\left(\frac{100\;ml}{100\;g\cdot brain}\right)\left(\frac{1}{\rho_{Voi}}\right)\left(\frac{K_{T}}{K_{A}}\right)\left(\frac{1-H_{A}}{1-H_{T}}\right)\max\left[k(t)\right]\\ =\frac{k_{H}}{\rho_{Voi}}\max\left[k(t)\right] \end{array}$$

(5) $$\begin{array}{l} CBV\;\left[\frac{ml}{100g}\right]\\ =\left(\frac{100\;ml}{100g\cdot brain}\right)\left(\frac{1}{\rho_{Voi}}\right)\left(\frac{K_{T}}{K_{A}}\right)\left(\frac{1-H_{A}}{1-H_{T}}\right)\int_{0}^{\infty }dt\;k(t)\\ =\frac{k_{H}}{\rho_{Voi}}\;\int_{0}^{\infty }dt\;k\left(t\right) \end{array}$$

*k*_*H*_ being a constant that groups together all the parameters needed to obtain absolute measurements of the perfusion parameters. $\left(\frac{K_{T}}{K_{A}}\right)\left(=0.136\right)$ is the tissue-to-artery concentration scale factor ratio, *H*_*A*_(= 0.45) is the assumed hematocrit in large arterial vessels, *H*_*T*_(= 0.25) is the assumed hematocrit in the capillary bed in the tissue, and ρ_*Voi*_(= 1.04 *g*/*ml*) is the apparent brain density. It should be noted that, CBV can be obtained via an alternative non-deconvolution method, relating the contrast agent in the tissue, *C*_*m*_(*t*), with the contrast agent in the AIF, *C*_*AIF*_(*t*) (Calamante et al., 1999, 2000; Konstas et al., 2009; Fieselmann et al., 2011),


(6) $$CBV\;\left[\frac{ml}{100g}\right]=\;\frac{k_{H}}{\rho_{Voi}}\left(\frac{\int_{0}^{\infty }dt\;C_{m}(t)}{\int_{0}^{\infty }dt\;C_{AIF}(t)}\right)$$

Finally, the MTT values are related to the CBF and CBV through the central volume theorem,


(7) $$\begin{array}{l} MTT\;\left[s\right]=\;\frac{CBV}{CBF} \end{array}$$

This equation holds true for the volume of interest, and it can be applied independently of the method used to calculate the CBV.

Due to being supported by a reliable mathematical model, CBF, CBV, and MTT are considered the three main perfusion parameters. However, there are other parameters that also provide important information outside those three main quantities. SR and PSR are two of those and they play important roles in the clinical diagnosis of GBM. These variables provide insightful information even when no underpinning mathematical model is present. Contrary to previous hemodynamic parameters, whose calculation requires time-consuming post-processing of the DSC-MRI images, obtaining SR and PSR is much faster and more straightforward. Following (Huhndorf et al., 2016) they can be calculated as,


(8) $$\{\begin{array}{c} SR\;\left[a.u.\right]=100\times \frac{S_{post}-S_{pre}}{S_{pre}}\\ PSR\;\left[a.u.\right]=100\times \frac{S_{post}-S_{\min}}{S_{pre}-S_{\min}} \end{array}$$

These two parameters only depend on the signal intensities at different times of the bolus passage through the tissue. *S*_*post*_ is the signal intensity at a time after the bolus arrival, usually 60 s after, *S*_*pre*_ is the signal intensity at baseline, before the bolus arrival to the tissue, and *S*_min_ is the minimum in signal, corresponding to the peak of the bolus. Restoring baseline signal intensity corresponds to a value of 100% on the PSR and SR maps.

### 2.2. Software implementation

This study presents an open DSC quantification tool for preclinical studies (Perfusion-Nobel). The implementation was carried out with Python (V 3.8.1), and the following libraries were used: NumPy (V 1.24.1) (Harris et al., 2020), SciPy (V 1.10.0) (Virtanen et al., 2020), Re (V Python 3.9) (Van Rossum, 2020), Matplotlib (V 3.6.2) (Hunter, 2007), Pillow (V 9.4) (Murray et al., 2023), OpenCV (V 4.7.0.68) (Bradski, 2000), Pydicom (V 2.3.1) (Mason, 2011), and IPython (V 8.8.0) (Perez, 2007).

**Figure 1:**
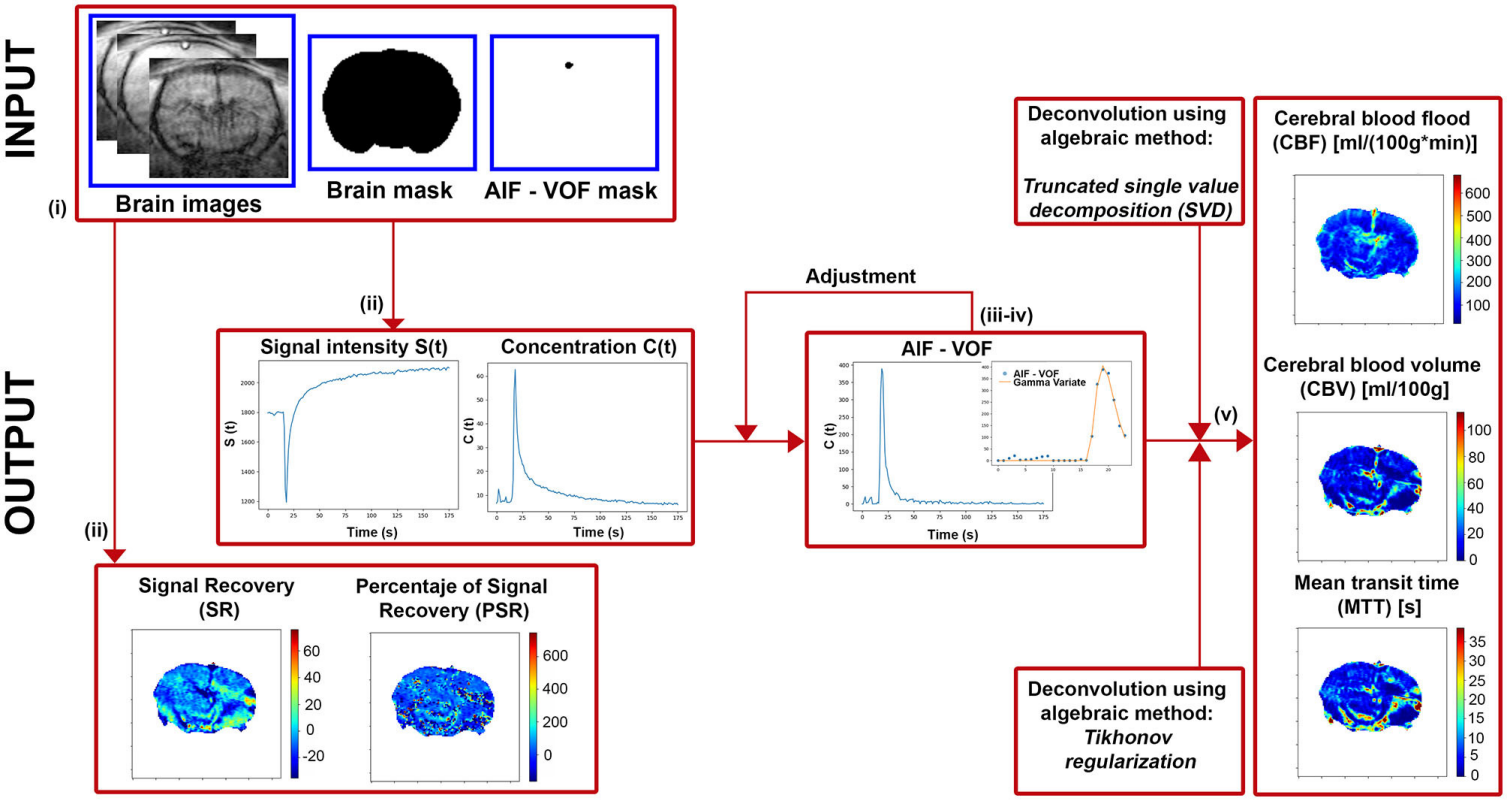
Diagram of the main steps implemented in our software to extract the perfusion-weighted images.

Modular construction has been used to implement the DSC quantification method. As a result, it is possible to easily replace existing stages or add new ones to the quantification workflow (for instance, new pre-processing algorithms or additional fitting models). The following is the processing workflow from the images acquired in the MR system (Figure 1):

**i)** The inputs requested by the software are: DSC-MRI in DICOM format, the brain mask, the AIF-VOF (PNG or NPY format) mask, and the frame number corresponding to the minimum signal intensity. The masking process should be done beforehand, either semi-manually by drawing the ROIs or using any automatic segmentation tool.
**ii)** DSC-MRI images are automatically pre-processed, reducing the noise by applying a 2D Gaussian Low Pass Filter (X and Y directions with a 5-pixel diameter and a standard deviation of 0.5 pixels). Then, the PSR and SR maps are computed, shown on screen, and automatically saved (TIFF format). Moreover, the mean image signal intensity curve (*S*(*t*)) and mean contrast concentration curve (*C*_*m*_(*t*)) are presented.
**iii)** Next, the AIF or VOF plot is shown, calculated as the mean contrast concentration curve in the AIF or VOF masked region. In brain perfusion, the mean cerebral or anterior carotid arteries are frequently utilized as reference vessels, corresponding to the highest signal intensity regions in T2^*^-weighted imaging. This function will be the global reference vessel for each pixel in the image.
**iv)** If we continue the analysis, it will display again the concentration curve corresponding to the reference vessel. Then, two values must be selected: (1) the arrival time of the contrast agent to the tissue and (2) the time after the first pass of the contrast agent through the tissue. These values are needed for the gamma variate function fitting to the concentration curve, and it will be shown on the screen. This process can be repeated for an accurate adjustment.
**v)** Finally, the deconvolution process will start, and the perfusion maps CBF, CBV, and MTT will be calculated, shown, and automatically saved (TIFF format). The deconvolution step can be done using TSVD or Tikhonov methods.

### 2.3. Validation against a data set

All experimental animal procedures were conducted under procedure numbers: 15011/2021/002, 15011/2021/003, 15011/2023/002, and 15011/2021/001 approved by the Animal Care Committee, according to European Union Rules and the Spanish regulation (2010/63/EU and RD53/2013). Animals were kept in a controlled environment at 22 ± 1°C and 60 ± 5% humidity, with 12:12 h light: darkness cycles, and were fed *ad libitum* with standard diet pellets and tap water. All surgical procedures and MRI studies were conducted under sevoflurane (Abbott Laboratories, IL, USA) anesthesia (3–4%) using a carrier 70:30 gas mixture of N_2_O:O_2_.

**Figure 2:**
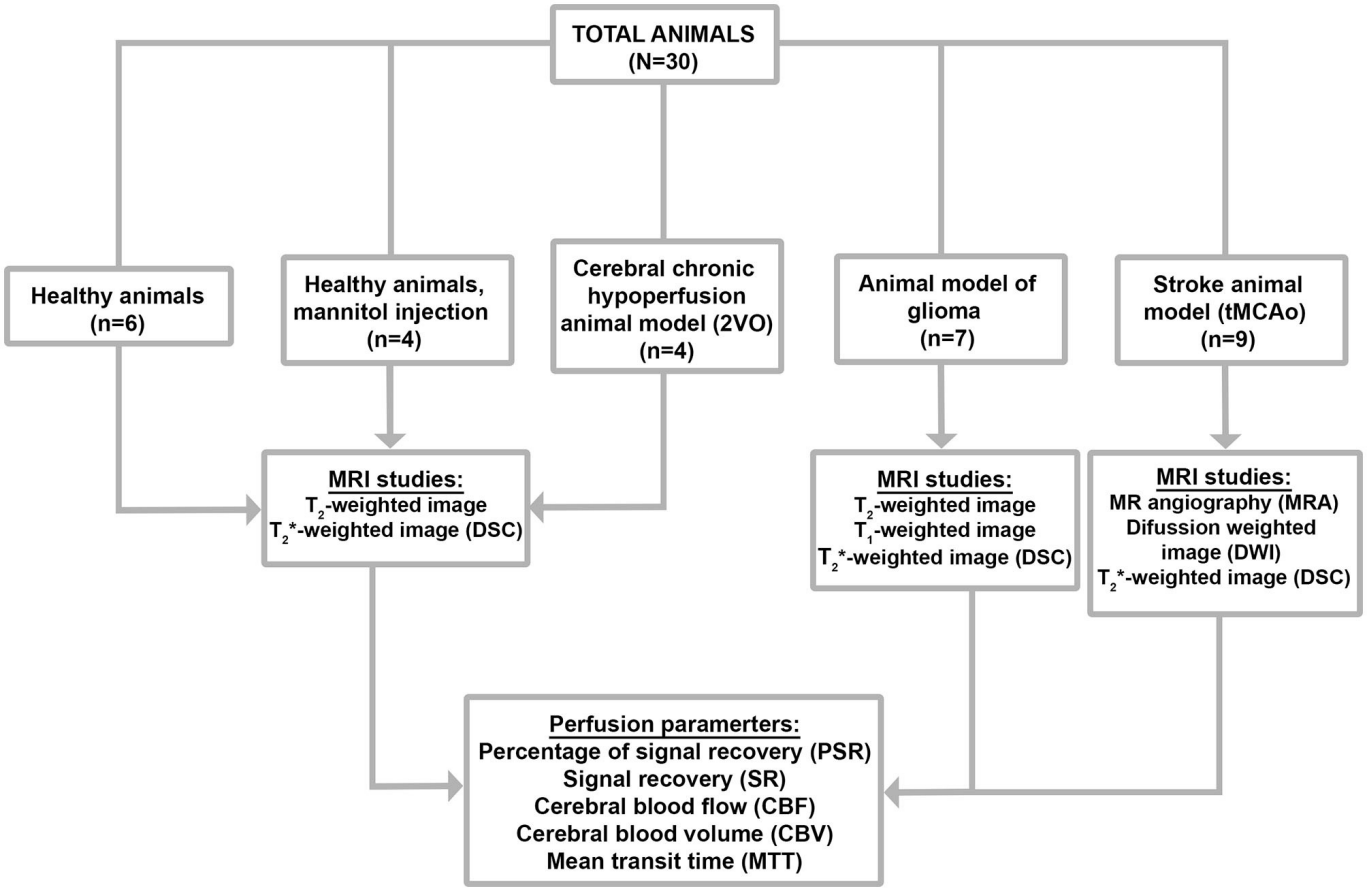
Flowchart of data set screening.

Thirty Sprague-Dawley rats with a weight between 250 and 350 g were used to test the developed program in animal models of different pathologies. Five groups of experiments are shown in Figure 2.

### 2.4. Healthy animals

We used healthy Sprague-Dawley (SD) rats with no surgery or treatment as the control healthy group (*n* = 6).

### 2.5. BBB disfunction model

Healthy rats (*n* = 4) were anesthetized, and X gr/Kg of mannitol [25% mannitol dissolved in isotonic 0.9% sodium chloride solution (B. Braun Medical SA, Barcelona, Spain)] was then intravenously injected (i.v.) in the lateral tail vein. MRI-DSC measurements were made starting 10 min after the mannitol injection (Duong et al., 2000).

### 2.6. Cerebral chronic hypoperfusion model

A group of rats (*n* = 4) were induced cerebral chronic hypoperfusion (CCH) by two-vessel occlusion (2VO), also known as permanent bilateral common carotid artery occlusion, as previously described by Cao et al., 2018. The animals were anesthetized, and the right common carotid artery was isolated and ligated with a 4-0, not absorbent suture through a ventral median incision in the cervical area. After 1 week, the left common carotid artery was ligated following the same protocol. MRI-DSC measurements were made 3 weeks after the surgery.

### 2.7. Glioblastoma multiforme model

GBM was induced in a group of rats (*n* = 7) by the implementation of the F98 cell line (Bulin et al., 2022). After anesthesia, animals were then mounted onto a stereotaxic frame. A midline incision was performed, and a burr hole was punctured using a 16-gauge needle. The burr hole coordinates were, using the bregma as a reference point, 1 mm anterior and 3 mm lateral to the right. Then 1 × 10^5^ cells suspended in a volume of 5 μl of non-supplemented DMEM were injected into the brain with a 26-gauge needle at a depth of 6 mm from the surface of the skull (injection rate of 0.2 ul/min). MRI-DSC measurements were evaluated 10 and 20 days after the surgery.

### 2.8. Ischemic stroke animal model

Regarding the stroke group (*n* = 9), transient focal ischemia was induced in rats by transient middle cerebral artery occlusion (MCAo) following surgical procedures previously described in Vieites-Prado et al. (2016). In brief, using 6-0 silk sutures, the right external carotid artery as well as the pterygopalatine artery of the internal carotid was ligated. A silicon rubber-coated size 4–0 monofilament (diameter 0.19 mm, length 23 mm; diameter with coating 0.37 ± 0.02 mm; coating length 3–4 mm) (Doccol Corporation, Sharon, MA) was inserted into the stump of the right common carotid artery and advanced into the internal carotid artery to 20 mm from the bifurcation to occlude the origin of the MCA. A laser Doppler flow probe (tip diameter 1 mm) attached to a PeriFlux 5000 Laser Doppler Flowmeter (Perimed AB, Stockholm, Sweden) was placed over the thinned skull in the MCA territory (4 mm lateral to bregma) to obtain a continuous measure of relative cerebral blood flow during the experiment Diffusion-weighted imaging (DWI), magnetic resonance angiography (MRA), and MRI-DSC measurements were made starting 20–30 min after the onset of MCA occlusion. The suture was removed 75 min after the occlusion.

### 2.9. DSC-MRI

All studies were conducted on a Bruker BioSpec 9.4 T MR scanner (horizontal bore magnet with 12 cm wide Bruker BioSpin) equipped with actively shielded gradients (440 mT m^−1^). Animals were imaged with a combination of a linear birdcage resonator (7 cm in diameter) for signal transmission and a 2 × 2 surface coil array for signal detection, positioned over the head of the animal, which was fixed with a teeth bar, earplugs, and adhesive tape. Animals were physiologically monitored throughout the MR imaging experiments. Transmission and reception coils were actively decoupled from each other.

DSC-MR images were acquired using ultra-fast gradient-echo methods (EPI-T2^*^) with the following parameters: 6.2 ms of echo time (ET), 1 s of repetition time (RT), number of repetitions (NRs) = 180, 1 average, 50 kHz spectral bandwidth (SW), flip angle (FA) of 90°, and 5 slices of 1.5 mm. Field of view (FOV) of 2.2 × 2.2 cm^2^, and a matrix size of 128 × 128, giving an in-plane resolution of 172 μm/pixel, implemented without fat suppression. The contrast agent was quickly administrated as a bolus 20 s after starting acquisition [intravenous (i.v.) bolus injection of gadolinium contrast agent (0.3 mmol/kg)]. The total DSC scan acquisition time was 3 min.

Brain volumetry study was also evaluated from T2-wi using a rapid acquisition with relaxation enhancement (RARE) sequence (axial and coronal orientations): with an ET = 11 ms, RT = 2.5 s, Rare Factor (RF) = 8, FA = 180°, NA = 3, SW = 37 KHz, 14 slices of 1 mm, 25.6 × 25.6 mm^2^ FOV, and a matrix size of 256 × 256 (isotropic in-plane resolution of 100 μm^2^/pixel).

To evaluate the status of MCAo in a non-invasive manner during occlusion in the stroke animal model, time-of-flight magnetic resonance angiography (TOF-MRA) was performed. The TOF-MRA scan was performed with a 3D FLASH sequence with TE = 2.5 ms, TR = 15 ms, FA = 20°, NA = 2, SW = 98 KHz, 1 slice of 14 mm, FOV = 30.72 × 30.72 × 14 mm^3^, and a matrix size of 256 × 256 × 58 (resolution of 120 × 120 × 241 μm^3^/pixel). Moreover, apparent diffusion coefficient (ADC) maps were obtained from diffusion-weighted image (DWI) using a spin echo-planar imaging sequence (DTI-EPI): ET = 26.91 ms; RT = 4 s; SW = 200 KHz; seven b-values of 0, 300, 600, 900, 1,200, 1,600, and 2,000 s/mm^2^; FA = 90°; NA = 4; 14 consecutive slices of 1 mm; FOV = 24 × 16 mm^2^, and a matrix size of 96 × 64 (isotropic in-plane resolution of 250 μm^2^/pixel).

### 2.10. Data analysis and statistics

We used region of interest (ROI) analysis to quantify the absolute maps CBV, CBF, MTT, and SR, PSR obtained in our new program. Next, through ImageJ software (Rasband W, NIH, Bethesda, MD, USA), we placed a circular ROI over each hemisphere cortex at the plane and a whole-brain ROI. For the ischemic stroke animal model, ROIs were situated in the core, penumbra region of the ipsilateral (IL) hemisphere, and cortex of the contralateral (CL) hemisphere. In the last group, the peripheral tumor area (tumor rim) and core ROIs were placed. Adjacent slices were measured to permit discrimination between intra- and inter-subject variance. To test whether the two slices can be considered samples of the same mean, a paired *t*-test was performed with the values from the two slices (*p* < 0.05). We generated Bland–Altman (BA) plots to compare our results (CBV, CBF, and MTT) with the literature data, where the horizontal axis represents the mean value [(our data + literature data)/2], and the vertical axis represents the difference; this method was used to compare two different measurement techniques (Giavarina, 2015). Data are presented as the mean ± 1.96^*^SD.

## 3. Results

A total of 30 animals were evaluated with our DSC-MRI quantification tool, and these results were compared in good agreement with values reported in the literature for rats. Table 1 reports our results, as well as a literature overview of absolute perfusion values obtained with different procedures, experimental conditions, rat strains, and post-processing methods.

**Table 1:** Literature review: absolute perfusion parameters CBV, CBF, and MTT in healthy rats, rats with a mannitol i.v. injection, rat brain hypoperfusion model, ischemic stroke rat model, and glioblastoma rat model.

**Technique**	**ROI**	**CBV** **(ml/100 g)**	**CBF (ml/100 g/min)**	**MTT** **(s)**	**Reference**
**Healthy rats**				
^1^MRI [^(a)^RSST1]	Whole brain parenchyma Cortex Striatum	3.29 ± 0.69 3.13 ± 0.91 2.92 ± 0.68	-	-	Perles-Barbacaru and Lahrech, 2007
^1^MRI [^(b)^pCASL] ^1^Autoradiograpy ^2^MRI [^(b)^pCASL] ^2^Autoradiograpy	Whole-brain parenchyma “ “ “	-	116 ± 14 111 ± 18 108 ± 12 109 ± 22	-	Larkin et al., 2019
^1^MRI [^(c)^CASL]	Right motor cortex (saline) Left motor cortex (saline) Right motor cortex (soman) Left motor cortex (soman)	-	150 ± 40 149 ± 41 132 ± 40 126 ± 36	-	Lee et al., 2021
^2^CT [^(d)^SRCT]	Left frontal cortex Right frontal cortex Left parietal cortex Right parietal cortex Whole brain	2.44 ± 0.57 2.44 ± 0.58 2.09 ± 0.38 2.10 ± 0.32 4.18 ± 1.06	154 ± 19 147 ± 17 129 ± 9 129 ± 19 215 ± 47	-	Adam et al., 2003
^2^MRI [^(e)^btASL]	Primary motor cortex Secondary motor cortex	-	-	2.00 ± 0.04 2.15 ± 0.06	Rouine et al., 2013
^1^MRI [^(f)^DSC]	Whole brain parenchyma Right cortex Left cortex	10.2 ± 1.7 7.2 ± 1.1 6.8 ± 1.2	104.4 ± 12.5 87.7 ± 6.7 91.3 ± 12.5	5.9 ± 0.2 4.8 ± 0.5 4.3 ± 0.4	This study
**Mannitol injection in rats**				
^1^MRI [^(g)^ASL] MRI [^(f)^DSC]	Motor cortex CL side Motor cortex IL side	-	93 ± 9 109 ± 10 > IL side	-	Tanaka et al., 2011
^1^MRI [^(f)^DSC]	Whole brain parenchyma Right cortex Left cortex	8.8 ± 2.0 6.1 ± 1.8 5.7 ± 1.4	66.6 ± 12.9 60.4 ± 14.8 58.9 ± 13.6	8.7 ± 0.4 5.8 ± 0.5 6.4 ± 0.5	This study
**Rat brain hypoperfusion model**				
^1^MRI [^(h)^CASL]	Sensory cortex (before) Sensory cortex (after)	-	214 ± 38.9 87 ± 14.9	-	Thomas et al., 2006
^1^MRI [^(h)^DCE]	Hippocampus sham Hippocampus 2VO	5.1 ± 0.8 4.5 ± 0.8	-	-	Livingston et al., 2020
MRI [^(f)^DSC]	Whole brain parenchyma Right cortex Left cortex	6.4 ± 2.2 4.2 ± 0.3 4.0 ± 1.2	53.5 ± 15.1 44.9 ± 11.3 46.2 ± 14.4	7.8 ± 0.6 6.0 ± 0.8 5.3 ± 0.4	This study
**Ischemic stroke rat model (MCAo)**				
^1^MRI [^(g)^ASL]	IL side CL side	-	31 ± 2.0 153 ± 4.6	-	Boisserand et al., 2017
^1^MRI [^(g)^ASL]	IL side CL side	-	4.3 ± 5.3 155 ± 50	-	Robertson et al., 2011
^2^MRI (Hydrogen Clearance)	IL side CL side	-	24 ± 6 114 ± 12	-	Cipolla et al., 2017
^3^MRI [^(g)^ASL]	IL side CL side	-	49 ± 4 135 ± 23	-	Reid et al., 2012
^1^MRI [^(f)^DSC]	IL side CL side	12.55 ± 5.31 21.1 ± 9.4	131.82 ± 81.4 244.69 ± 173	5.43 ± 0.63 6.02 ± 1.78	Tsai et al., 2021
^1^MRI [^(b)^pCSAL]	IL motor cortex CL motor cortex	-	104 ± 28 170 ± 21	-	Baskerville et al., 2012
^2^MRI [^(f)^DSC]	IL side CL side	2.34 ± 0.35 8.43 ± 0.85	-	15.91 ± 2.38 7.31 ± 0.79	Zhang et al., 2014
^1^MRI [^(f)^DSC]	CL side IL side (penumbra) IL side (core)	12.0 ± 5.6 9.3 ± 4.2 6.2 ± 3.1	75.1 ± 21.1 47.8 ± 13.4 17.2 ± 9.0	9.6 ± 2.5 12.2 ± 3.9 20.4 ± 6.9	This study
**Glioblastoma rat model**				
^2^MRI [^(f)^DSC]	Tumor CL side	5.0 ± 0.5 2.0 ± 0.5	10 ± 4 30 ± 11	10 ± 3 4.7 ± 0.7	García-Palmero et al., 2013
^2^MRI [^(f)^DSC]	Tumor	3.6 ± 1.5 5.0 ± 2.5 4.5 ± 0.5 5.6 ± 2.5	52.5 ± 12 80 ± 11 72.8 ± 15 66.2 ± 14	3.8 ± 1 4.5 ± 0.7 3.8 ± 1.5 4.9 ± 2.5	Stokes et al., 2014
^4^MRI [^(i)^CASL]	Tumor	-	64 ± 12 55 ± 10	-	Gonawala et al., 2018
^4^MRI [^(b)^pCSAL]	Tumor Periphery tumor Healthy tissue Tumor Periphery tumor Healthy tissue	-	145.92 ± 29.82 121.99 ± 25.24 97.26 ± 18.94 94.21 ± 34.58 81.20 ± 27.96 95.11 ± 15.05	-	Clément et al., 2021
^2(j)^CT	Tumor Healthy tissue	5.2 ± 1.5 3.5 ± 0.5	125.9 ± 2.5 124 ± 3.5	-	Qi et al., 2021
^1^MRI [^(f)^DSC]	CL side IL side (periphery) IL side (core)	4.9 ± 1.6 1.4 ± 0.7 3.4 ± 2.1	36.8 ± 15.3 40.7 ± 33.4 10.3± 4.6	8.1 ± 2.1 2.2 ± 0.9 16.3 ± 8.4	This study
^1^MRI [^(f)^DSC]	CL side IL side (periphery) IL side (core)	9.4 ± 2.4 4.1 ± 3.0 1.2 ± 0.3	118.8 ± 47.1 203.5 ± 18.2 104.1 ± 19.8	9.6 ± 2.7 12.2 ± 3.9 0.67 ± 0.1	This study

^1^Sprague-Dawley, ^2^Wistar, ^3^Wistar Kyoto, ^4^Nude rats.

^(a)^Rapid steady-state T_1_ (RSST_1_), ^(b)^ pseudo-continuous arterial spin labeling (pCASL), ^(c)^ continuous arterial spin labeling (ASL), ^(d)^ synchrotron radiation computed tomography (SRCT), ^(e)^bolus-tracking arterial spin labeling (btASL), ^(f)^ dynamic susceptibility contrast (DSC), ^(g)^ arterial spin labeling (ASL), ^(h)^ dynamic contrast-enhanced (DCE), ^(i)^ continuous arterial spin labeling (CASL), and ^(j)^ computed tomography (CT).

**Figure 3:**
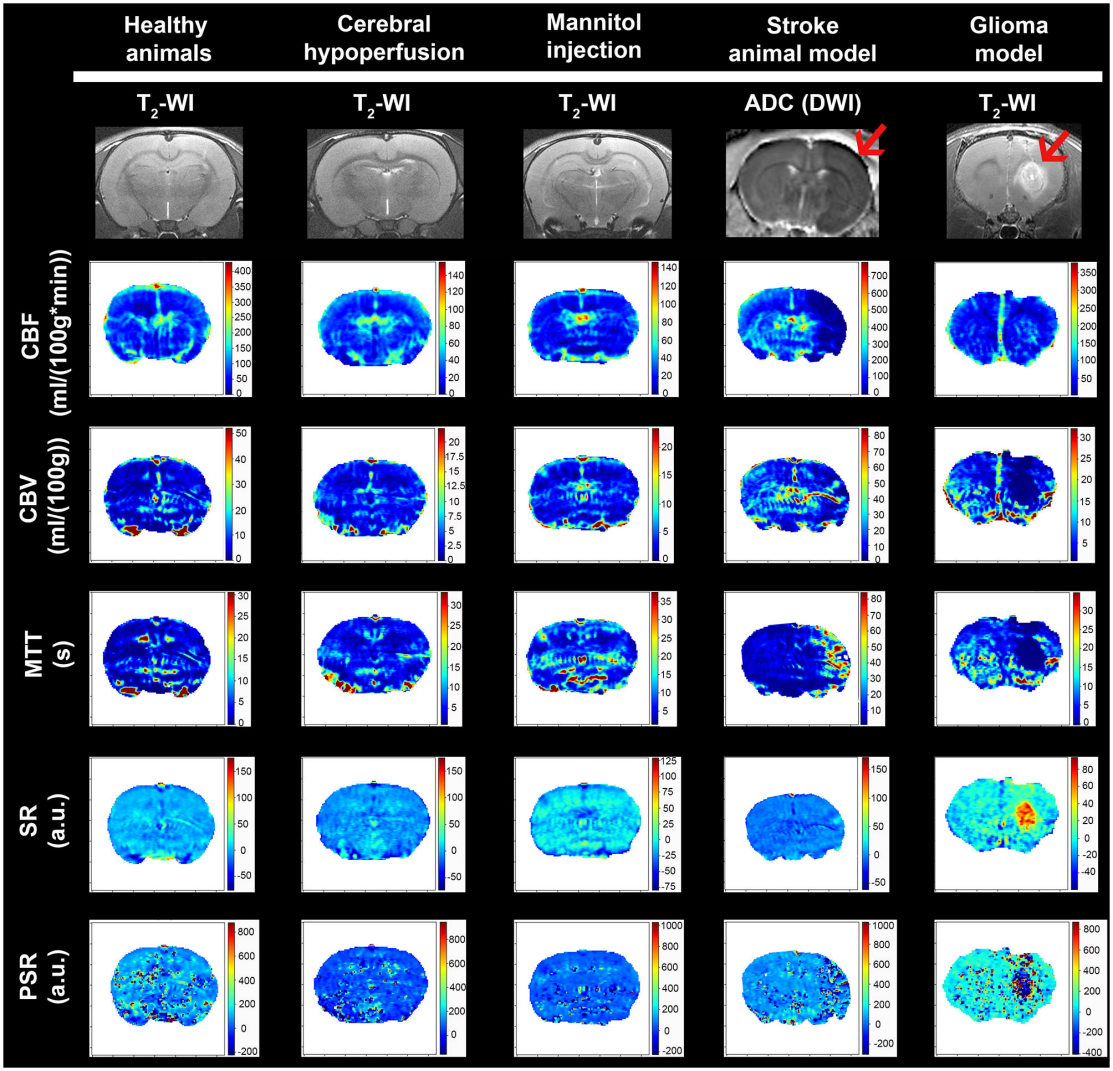
Examples of mean cerebral l blood flow (CBF), cerebral blood volume (CBV), mean transit time (MTT), signal recovery (SR), and percentage signal recovery (PSR) images generated with our tool for the different animal models. Anatomic MRI of the rat brain model used is detailed with T2-wi or DWI (ADC) (red arrows indicate the ischemic lesion and presence of glioma).

Our analysis has been developed as a Python script and has been published under a free software license (GNU GPL). Source code and binary downloads are available at https://github.com/MRI-NOBEL/Perfusion-NOBEL. Our tool successfully generated and displayed, in common animal models in neuroscience, all the perfusion parametric images: CBF, CBV, MTT, including PSR, and SR (Figure 3). Moreover, the anatomic details of the rat brain model used have been represented by means of a T2-wi or DWI (ADC) during the MCAo in the axial orientation (Figure 3). Regions of impaired flow under ischemic conditions and tumor influence areas can be delineated. Two distinct regions can be detected during the acute phase of stroke: an ischemic core that is severely and irreversibly damaged, and a penumbra region defined as ischemic tissue that is functionally impaired and at risk of infarction but has the potential to be salvaged (Boisserand et al., 2017). By means of hemodynamic variables, it is possible to accurately differentiate the core and peripheral tumor areas (Aprile et al., 2015). However, changes associated with chronic cerebral hypoperfusion or temporary BBB dysfunction models are only appreciable due to the quantitative values obtained.

### 3.1. Quantification of absolute perfusion parameters

**Figure 4:**
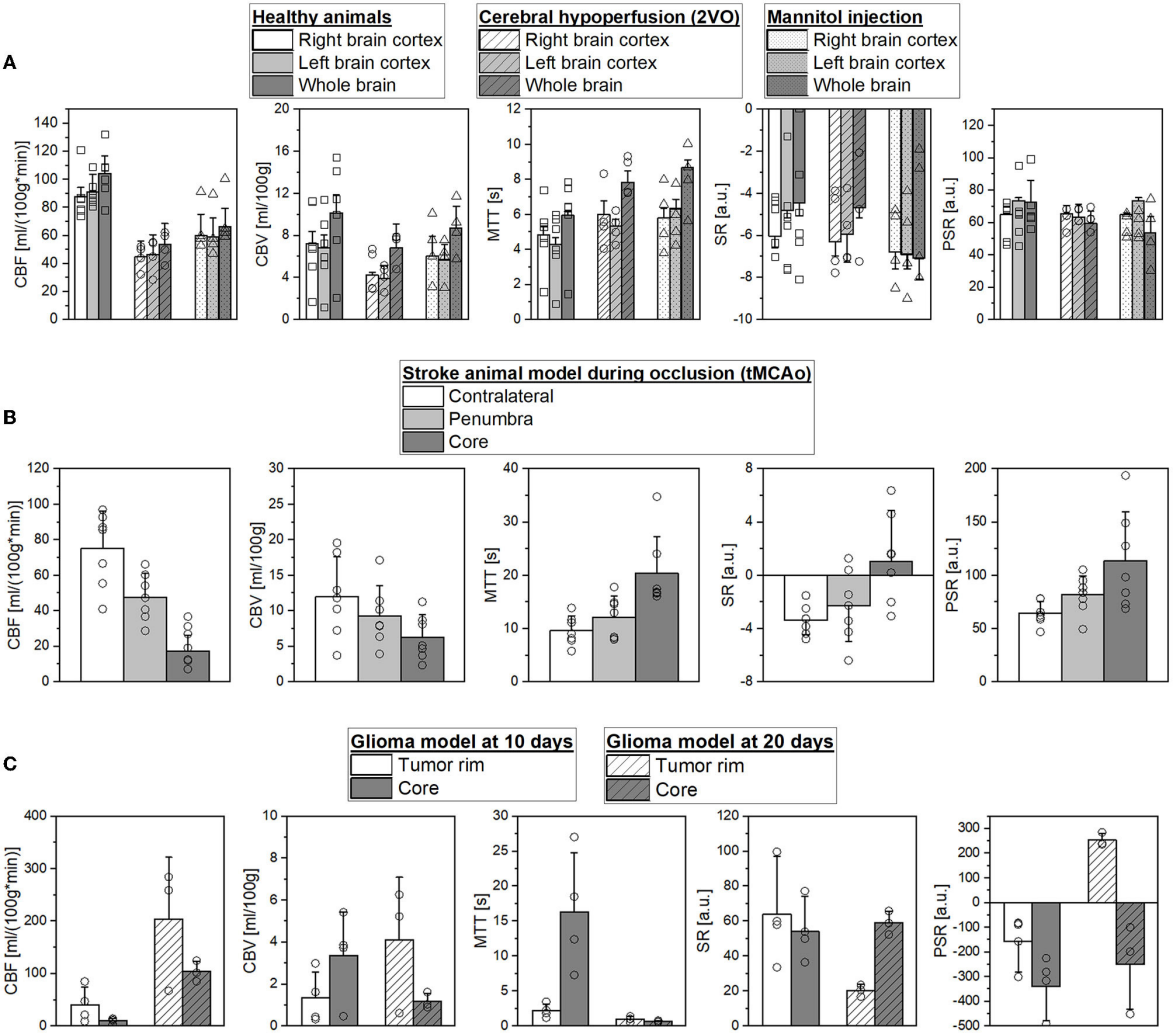
**(A)** Comparison of mean cerebral blood flow (CBF), cerebral blood volume (CBV), mean transit time (MTT), signal recovery (SR), and percentage signal recovery (PSR) in selected regions of interest (ROIs) of healthy SD rats, during hypoperfusion model, and mannitol injection. **(B)** Mean CBF, CBV, MTT, SR, and PSR in contralateral hemisphere, penumbra, and core ROIs during occlusion in an ischemic stroke animal model. **(C)** Mean CBF, CBV, MTT, SR, and PSR in the peripherical and core ROIs for glioblastoma model at 10 and 20 days after the surgery.

Changes in hemodynamic parameters were compared using left, right, and whole-brain ROIs between healthy animals and animals subjected to chronic hypoperfusion or temporal BBB dysfunction (Figure 4A). For all regions, CBF decreases by ~49–35.6% in both models compared to healthy animals. The same is for the CBV, although the reduction is only 32–13.7%, respectively. Mean MTT increased in all ROIs by ~40.7%, and SR or PSR shows a lower capacity to discriminate changes in these models.

Figure 4B illustrates the hemodynamic parameter evolution in a stroke animal model during MCAo. As can be seen, we found a CBF reduction of 36.4% and 77.2% in the penumbra and core regions in accordance with previous studies (Robertson et al., 2011; Reid et al., 2012; Cipolla et al., 2017), respectively [75.1 ± 21.1 vs. 47.8 ± 13.4 vs. 17.16 ± 9.0 ml/(100g^*^min)]. Moreover, CBF decreases by ~22.5 and 47.5% in both regions (12.0 ± 5.6 vs. 9.3 ± 4.2 vs. 6.3 ± 3.2 ml/100 g). Due to the absence of vascularization in these regions, the MTT values show an increase of 26.1 and 111.5% in the penumbra and core (9.6 ± 2.7 vs. 12.1 ± 3.9 vs. 20.3 ± 6.9 s). We found that SR and PSR have also the capacity to accurately differentiate the core from the other brain regions: CL (−3.3 ± 1.1%, 64.3 ± 11.1%), penumbra region (−2.3 ± 2.6%, 81.7 ± 18.1%), and lesion core (1.1 ± 3.8%, 113.5 ± 46.1%).

Parameters of tumor perfusion were validated in a preclinical GBM model at 10 and 20 days after the surgery known to produce different levels of vascularization (Figure 4C). We can appreciate that it is possible to accurately identify core and peripheral tumor regions at both time points. The CBF values in the tumor core were reduced at 10 and 20 days to 74.5 and 48.8%, respectively. At 10 days, animals showed increased CBV and MTT values in the core compared to the tumor rim (3.4 ± 2.1 vs. 1.3 ± 1.2 ml/100 g, and 16.3 ± 8.5 vs. 2.2 ± 0.9 s). However, at 20 days of CBV and MTT, a change in behavior is detected (1.2 ± 0.4 vs. 4.1 ± 3 ml/100 g, and 0.67 ± 0.1 vs. 1 ± 0.43 s). These results may reflect the heterogeneous structure of the tumor regions, and the time evolution probably also contributes to this heterogeneity. As previously described, SR and PSR maps provided a GBM spatial distribution and add valuable diagnostic information. At 20 days, we found SR core 59.1 ± 6.7% vs. tumor rim 20.4 ± 3.4%, and PSR core −249.4 ± 181.1% vs. tumor rim 253.5 ± 27.3%.

### 3.2. Bland–altman analysis

**Figure 5:**
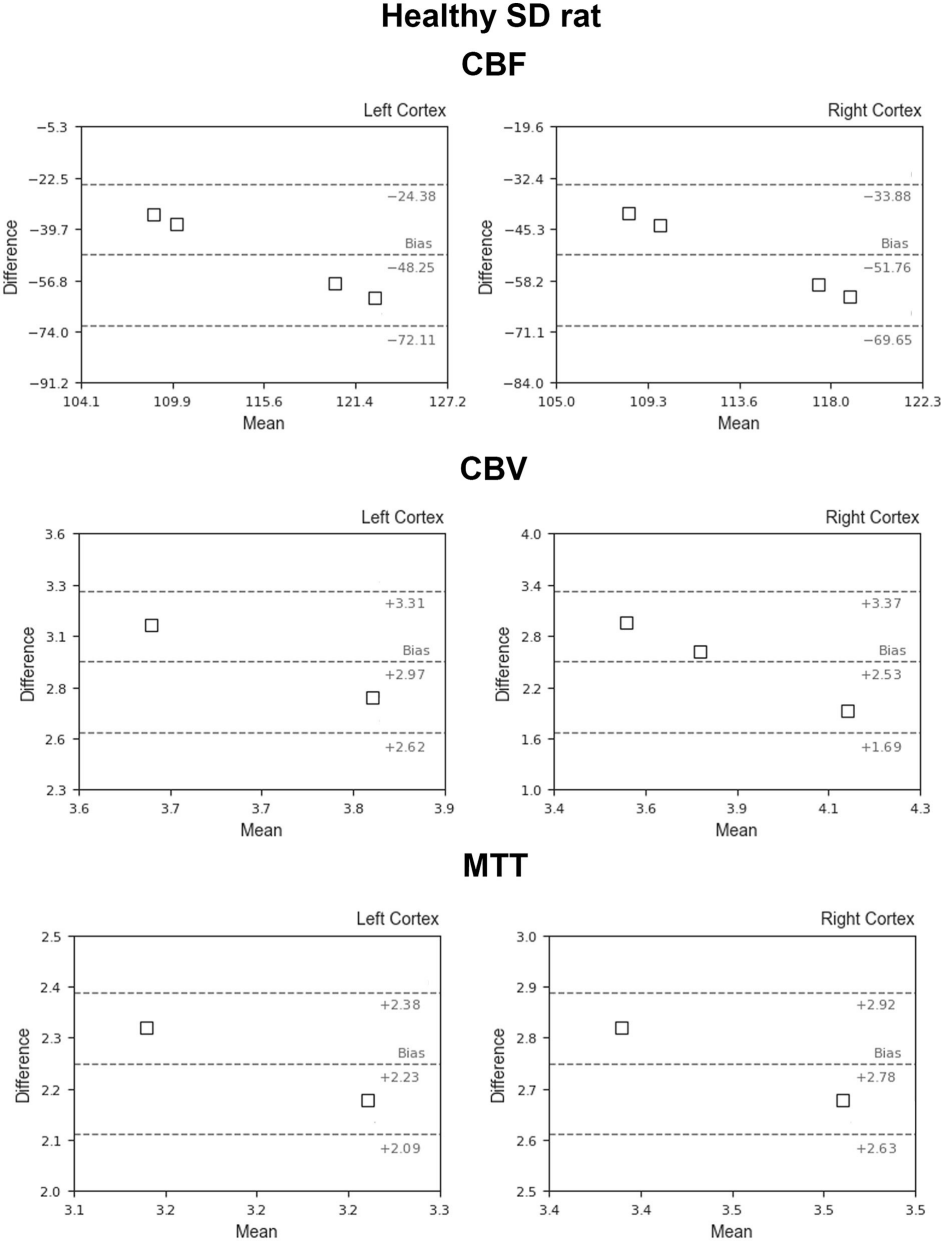
Bland–Altman plots for CBV, CBF, and MTT for our results vs. literature data regarding SD healthy rats (Adam et al., 2003; Perles-Barbacaru and Lahrech, 2007; Rouine et al., 2013; Lee et al., 2021). Dashed lines represent the bias, +95% (upper line) and −95% (lower line) of the limits of agreements.

**Figure 6:**
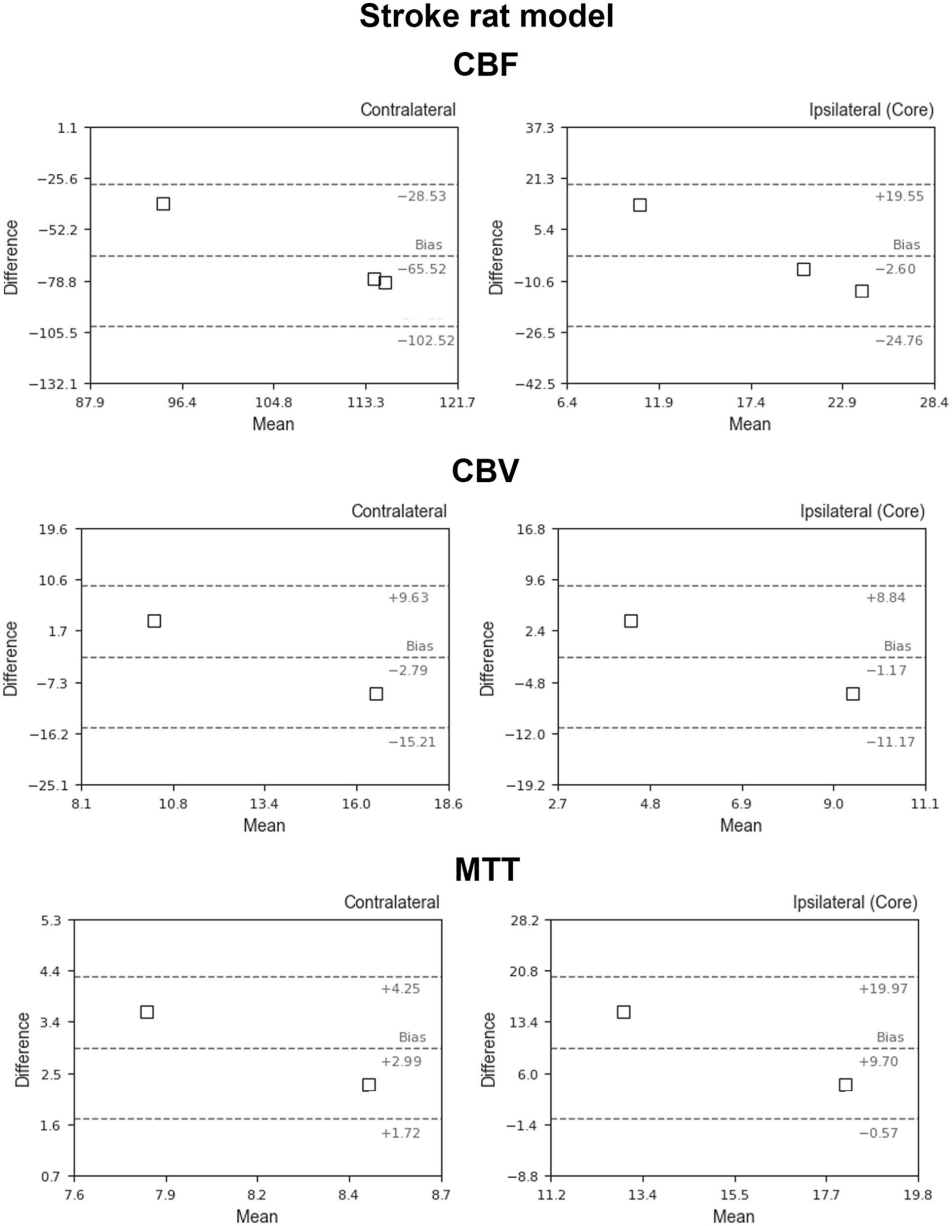
Bland–Altman plots for CBV, CBF, and MTT for our results vs. literature data regarding the ischemic stroke animal model (Thomas et al., 2006; Zhang et al., 2014; Boisserand et al., 2017; Livingston et al., 2020; Tsai et al., 2021). Dashed lines represent the bias, +95% (upper line) and −95% (lower line) of the limits of agreements.

**Figure 7:**
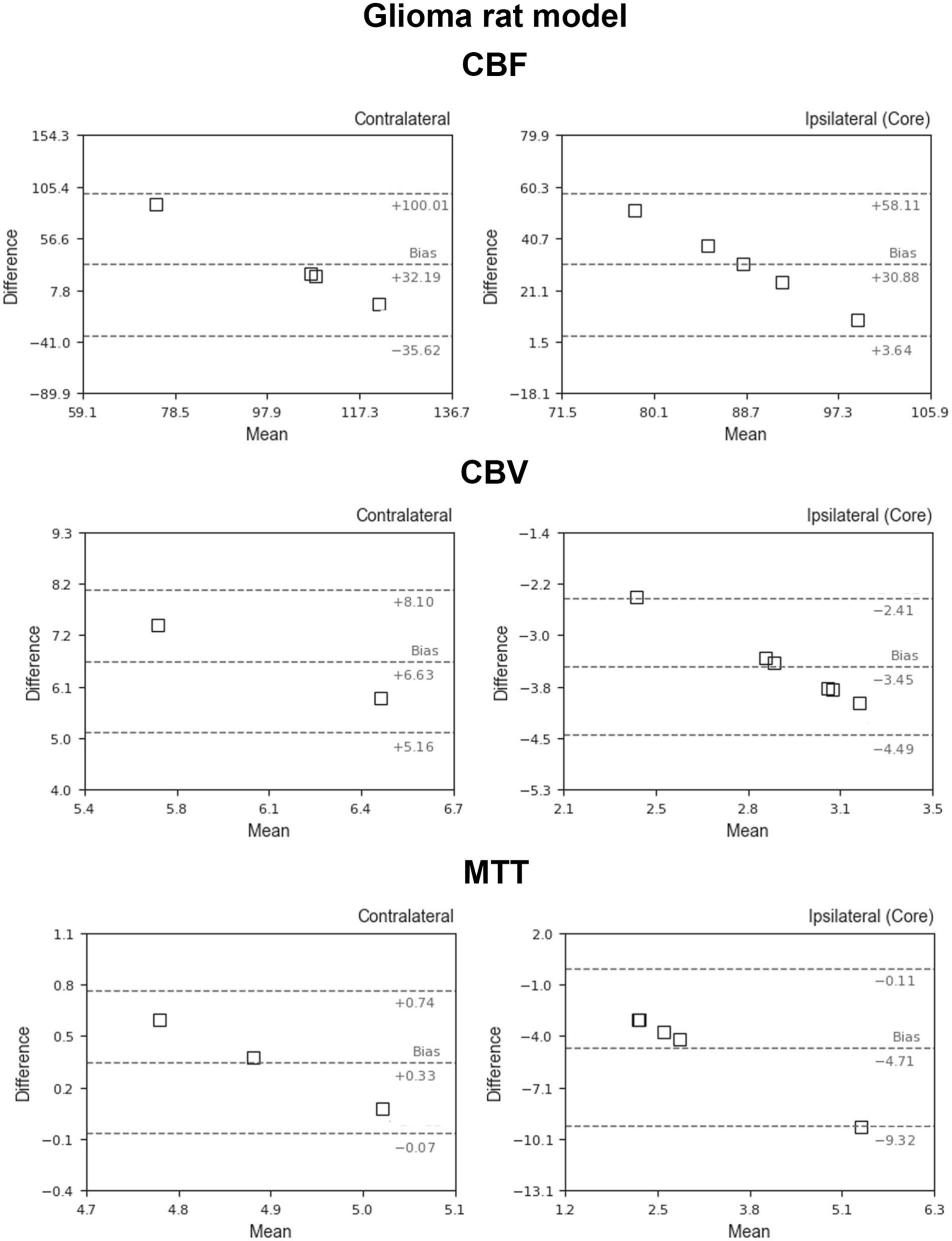
Bland–Altman plots for CBV, CBF, and MTT for our results vs. literature data regarding the glioblastoma animal model (García-Palmero et al., 2013; Stokes et al., 2014; Gonawala et al., 2018; Clément et al., 2021). Dashed lines represent the bias, +95% (upper line) and −95% (lower line) of the limits of agreements.

Figure 5 shows the BA plots for our data and healthy SD rats, corresponding to the left and right cortex. We observed low mean differences for CBV (2.5 ml/100 g) and MTT (2.7 s) variables and large differences for CBF [49.9 ml/(100 g^*^min)]. Regarding the ischemic stroke model, we compared the lesion core and CL cortex, for our results and literature data (Figure 6). The BA plot highlighted low mean values of bias for CBF [2.6 ml/(100 g^*^min)], CBV (1.17 ml/100 g), and MTT (9.7 s) for the core lesion. We found the same for CBV (2.8 ml/100 g) and MTT (3 s) in the CL cortex, but not for CBF [65.2 ml/(100 g^*^min)]. Regional BA analysis of the tumor core and CL cortex is illustrated in Figure 7. We observed low mean discrepancies for CBV (−3.45 and 6.63 ml/100 g) and MTT (−4.71 and 0.73 s) variables, and large discrepancies for CBF [30.9 and 32.2 ml/(100 g^*^min)] both in the tumor core and in the CL cortex.

## 4. Discussion

The absolute quantification of perfusion-weighted parameters is useful for preclinical research studies of different neurological diseases. The integrity of the BBB is critical to normal brain function, and the perfusion variables allow the detection of brain flow dysregulation areas as the origin of future brain events. Recently, cerebrovascular dysfunction has emerged as an early manifestation of Alzheimer's disease with a direct impact on the evolution and clinical expression of dementia. It has been shown that mice expressing mutated tau exhibit a selective suppression of neural activity-induced CBF increases that precedes tau pathology and cognitive impairment (Warpechowski et al., 2023). Regarding stroke, these parametric maps help to diagnose and predict the final cerebral ischemic stroke volume identifying core and penumbra regions (Demeestere et al., 2020). In addition, it is also a valuable diagnostic tool for tumor grading that may contribute to a better understanding of tumor evolution and characterize the vascularization of the tumor rim and core (Aprile et al., 2015; Huhndorf et al., 2016).

Regarding preclinical MRI data processing, there is variability in the programs for computing the perfusion variables that use different methods for the analysis, both regarding the mathematical modeling and the protocols of image acquisition. Moreover, most of them have closed access, limited to the institution responsible for the software development (Gordaliza et al., 2015; Huhndorf et al., 2016; López-Larrubia, 2018; Hartmann et al., 2020; Tsai et al., 2021). This issue is present even in human health, causing concerns about the accuracy of software quantitative perfusion parameters. Therefore, the obtained hemodynamics parameters cannot be easily compared between different studies, and perfusion-weighted methods remain an active area of research.

In this study, the DSC quantification tool designed performs the basic mathematical steps to generate the main hemodynamic parametric maps CBV, CBF, MTT, including PSR, and SR solving the indicator dilution model through deconvolution-based methods, being implemented in both TSVD and Tikhonov regularization. Furthermore, it contains an additional processing step, fitting the data to a gamma function in order to remove the effects of both noise and contrast recirculation. The tool works as Python script and has been published under a free software license with a modular architecture to allow own improvements or new pre-processing algorithms (filters, alignments, or image corrections). The parametric maps were obtained with an acceptable signal-to-noise ratio (SNR) to identify the small blood signal with a spatial resolution of 0.172 × 0.172 × 1.5 mm^3^/pixel and can be stored using standard image formats to carry out the posterior analysis. In general, SR and PSR maps appear noisier. However, these parameters can be rapidly acquired, and they do not require complex mathematical post-processing steps and help to characterize, e.g., tumor vasculature between the core and periphery over time.

It is important to note that the absolute quantification of hemodynamic parameters requires the capability of detecting signal variations in or adjacent to a large vessel, usually the paraclinoid or middle cerebral arteries. However, due to the low spatial resolution, image-based determination of AIF in rodents is challenging and very difficult to achieve. This limitation usually overcome using different techniques, though it may be useful to perform a semiquantitative quantification using laboratory arbitrary units (a.u.) or normalization of the obtained data. There are also more complex techniques that automatically determine AIF based on, among other things, the detection of pixels with the greatest contrast enhancement in various regions of interest, the use of clustering techniques, or the creation of physical models for the echo-planar signal intensity from an artery (Ostergaard et al., 1996; Wu et al., 2003; López-Larrubia, 2018). In this study, we provide the possibility of semi-automatic AIF or VOF adjustment with real curve monitoring for an accurate AIF-pixel selection.

To the extent of our knowledge, there is no standard recommendation software for preclinical perfusion with a universally accepted rating. Thus, for the validation and examination of our tool, we used diverse data sets from different rat models of brain diseases: i) temporal BBB dysregulation animals, ii) cerebral CCH model, iii) ischemic stroke, and vi) GBM. In general, our software yielded excellent results in terms of agreements for the expected brain regional analysis based on the animal model addressed.

It has been reported that in the cerebral chronic hypoperfusion model 2VO, 2 weeks after the surgery, the CBF recovers to 55–65% of the control level (Cao et al., 2018). Our results showed 51% of CBF getting back 3 weeks after the surgery. Regarding the mannitol group, a previous study described that CBF increased predominantly in the hemisphere in which mannitol was injected. In our study, an alternative experimental model was employed, mannitol was i.v. injected in the tail as a recognized and reliable procedure to temporal modulate the BBB permeability. We found that CBF decreases by ~35.6% related to healthy animals, while CBV and MTT values remained similar or increased at 10 min after mannitol injection (Duong et al., 2000; Tanaka et al., 2011). As has been previously described (Robertson et al., 2011; Reid et al., 2012; Cipolla et al., 2017), ischemic core and penumbra areas were calculated based on a CBF reduction compared with the equivalent CL hemisphere ROI (36.4 and 77.2%). From these regional definitions, the CBV and MTT maps obtained are in agreement with the expected metabolic values for these regions due to the limited vascularization. The preclinical tumor model at 10 and 20 days after the surgery allowed us to compare the time level vascularization. Previous studies determined that 4–9 days after tumor detection, the capillary permeability significantly increases in the core (Stadlbauer et al., 2015; Choi et al., 2016; Huhndorf et al., 2016). This increase in capillary permeability most likely reflects the beginning of necrosis within the tumor core that will continue to evolve over time. The regional analysis obtained in our study reveals that at 10 days, areas of increased CBV are mostly found at the core (3.4 ± 2.1 vs. 1.3 ± 1.2 ml/100 g). However, we can appreciate that this behavior is reversed after 20 days (1.2 ± 0.4 vs. 4.1 ± 3 ml/100 g), which reflects that the nucleus has necrotized and there is a vascular proliferation at the tumor rim.

The BA plot was also used to describe the agreement between our perfusion quantitative analyses and literature data regarding healthy rats, stroke, and GBM models. In general, the agreement for CBV and MTT is higher than for CBF. Possible explanations are the high CBF sensitivity errors due to the localization and delineation of ROIs, and the microvasculature mechanism effects of the mathematical tracer kinetic models. The higher sensitivity of DSC-MRI to susceptibility artifacts, an effect known and already studied (Maral et al., 2020), could also be another reason, being supported by the higher sensitivity to artifacts observed in the CBV and MTT maps, compared to the corresponding CBF map.

Finally, Table 1 reports a literature overview of absolute perfusion values obtained with different procedures, experimental conditions (including several rat strains), and different post-processing methods. Although it is difficult to compare, all hemodynamic parameters reported from the literature are in the same range as our results. In general, few studies assess CBF, CBV, and MTT simultaneously, and many studies show high deviations probably as a result of the regional analysis developed. As it can be appreciated, arterial spin labeling (ASL) MR perfusion is another technique commonly used that does not require intravenous administration of contrast. By using arterial blood water protons that have been magnetically labeled as endogenous tracers, this non-invasive and non-ionizing MRI technique assesses tissue perfusion (blood flow). Different techniques have been described to achieve ASL perfusion: i) pulsed (PASL), ii) continuous (CASL), iii) pseudo-continuous (PCASL), and iv) velocity-selective ASL (VS-ASL). However, the parameter most commonly derived in these protocols is CBF because CBV and MTT are difficult to reliably obtain, and this method usually presents low SNR and long acquisition time.

## 5. Conclusion

In order to facilitate the use and comparison of perfusion-weighted imaging in preclinical studies, we provide an open-source DSC post-processing software package. This software allows the calculation of several key quantitative perfusion parameters, such as CBF, CBV, and MTT, including SR and PSR maps from semi-automatic AIF or VOF adjustment. The open-source computational steps allow for possible improvements or pre-processing algorithms for other situations. The results obtained in diverse data sets of brain disease models in rats are consistent and in good agreement with values and behavior reported in the literature.

## Data availability statement

The raw data supporting the conclusions of this article will be made available by the authors, without undue reservation.

## Ethics statement

The animal study was reviewed and approved by 15011/2021/002, 15011/2021/003, 15011/2023/002, and 15011/2021/001.

## Author contributions

JC, RI-R, PH, and MA-A: organization and design of the study. JC, RI-R, PH, MA-A, SF-R, and GF-C: manuscript drafting. AO, AS-V, MB-B, MP-M, AF, JP, and AM: supervision, review, and critique. MP-M, EL-A, SF-R, and AS-V: data acquisition. All authors read, reviewed, and agreed upon the manuscript version.
